# Recent Advances in Molecular Technologies and Their Application in Pathogen Detection in Foods with Particular Reference to *Yersinia*

**DOI:** 10.4061/2011/310135

**Published:** 2011-10-29

**Authors:** Jin Gui, Isha R. Patel

**Affiliations:** ^1^College of Management and Technology, Walden University, 155 Fifth Avenue South, Minneapolis, MN 55401, USA; ^2^Division of Molecular Biology, Office of Applied Research and Safety Assessment, Center for Food Safety and Applied Nutrition, U.S. Food and Drug Administration, 8301 Muirkirk Road, MOD 1 Facility, Laurel, MD 20708, USA

## Abstract

*Yersinia enterocolitica* is an important zoonotic pathogen that can cause yersiniosis in humans and animals. Food has been suggested to be the main source of yersiniosis. It is critical for the researchers to be able to detect *Yersinia* or any other foodborne pathogen with increased sensitivity and specificity, as well as in real-time, in the case of a foodborne disease outbreak. Conventional detection methods are known to be labor intensive, time consuming, or expensive. On the other hand, more sensitive molecular-based detection methods like next generation sequencing, microarray, and many others are capable of providing faster results. DNA testing is now possible on a single molecule, and high-throughput analysis allows multiple detection reactions to be performed at once, thus allowing a range of characteristics to be rapidly and simultaneously determined. Despite better detection efficiencies, results derived using molecular biology methods can be affected by the various food matrixes. With the improvements in sample preparation, data analysis, and testing procedures, molecular detection techniques will likely continue to simplify and increase the speed of detection while simultaneously improving the sensitivity and specificity for tracking pathogens in food matrices.

## 1. Introduction

The genus* Yersinia* mainly includes animal pathogens, but animals can transmit disease to humans through direct or indirect contact [1]. Symptoms of illness can include diarrhea, vomiting, abdominal pain, and fever. There are three species within the genus *Yersinia* that are pathogenic for humans: *Yersinia enterocolitica, Yersinia pseudotuberculosis*, and *Yersinia pestis*. All these species have evolved with different clinical symptoms. *Y. enterocolitica* infections have been observed all over the world, but appear to be more common in Europe, especially in some Scandinavian regions, with much lower rates in the United States [2]. Food has often been suggested to be the main source of yersiniosis. Enteropathogenic *Yersinia*, *Y. enterocolitica, *and *Y. pseudotuberculosis, *entering the human body in contaminated food invade the M cells of the Peyer's patches [3]. The process and its effect on the host cell are driven by a large array of virulence factors that are deployed under genetic and environmental regulation. *Y. enterocolitica *can be categorized by biotype. Biotype 1A strain is considered as nonpathogenic, while 1B strain is considered as high-pathogenic, and biotypes 2, 3, 4, and 5 strains are considered as low-to-moderate pathogenic. The pathogenic phenotype can be differentiated due to the virulence-associated genes identified in these strains. 

 In the event of foodborne disease outbreaks, rapid identification of foodborne pathogens rely on the speed and simplicity of the detection method, which are critical for early detection and quick response [4]. The new advancement of high-throughput OMICS technologies provides scientists with the means to identify the agent and attribute it to a specific source of pathogenic *Yersinia* in food systems [5].

## 2. Current Advances in Detection Methods

One of the most challenging issues in food safety is the detection of foodborne pathogens. Since the infectious dose of many pathogens is as low as a few cells or particles [6], the sensitivity of the diagnostic tool becomes essential. In fact, the detection of pathogens in nonprocessed or minimally processed foods is not easy. Such foods are not sterile; the native microflora in such foods can mask the presence of a pathogen by interfering with isolation [7]. Thus, more sensitive and reliable detection methods have been developed in accordance with the advancement of molecular and biochemical technologies.

Isolation of *Y. enterocolitica* from clinical, food, and environmental samples can be challenging primarily due to the difficulty of growing *Y. enterocolitica in vitro *[8]*. *Traditional culture-dependent methods have several limitations, such as long incubation steps, lack of identification between species, and lack of discrimination between pathogenic and nonpathogenic strains [8, 9]. 

Numerous molecular techniques have emerged, that offer the advantage of speed along with specific and sensitive detection [10, 11]. Due to the relative simplicity, rapidity, reliability, and sensitivity, DNA-based detection technology plays an important role and provides detection methods in the form of next-generation sequencing [12], microarray [13], fluorescent in situ hybridization (FISH) [14], polymerase chain reaction (PCR) [15], molecular beacon technology [16], and many others. DNA testing is now possible on a single molecule, and high-throughput analysis allows thousands of detection reactions to be performed at once, thus allowing a range of characteristics to be rapidly and simultaneously determined. Some of the current molecular detection methods not only can be performed in the laboratory or clinical settings but also can be run at the observation site, such as on the farm or in the field, in the form of “all-in-one” kits [17, 18]. 

### 2.1. Genome Sequence

The release of the complete genome sequence of *Y. enterocolitica *strain 8081 provided important insights into the pathology of this bacterium [19]. There are 18 completed and over 160 incomplete *Yersinia* strains past and ongoing *Yersinia* genome sequencing projects (http://www.ncbi.nlm.nih.gov/genomes/lproks.cgi/) including *Y. pestis* strain CO92 [20] and *Y. pseudotuberculosis* strain IP 31758 at J. Craig Venter Institute/The Institute for Genomic Research [21]. These sequencing projects will enable the study of the evolution of the pathogenic changes in each species as they have adapted to new environmental surroundings. The information gathered from the genome sequences of the three major pathogenic *Yersinia* species will allow the development of a cross-species microarray for pathogenic *Yersinia* and will lead to invaluable insights into how the enteropathogens are adapted to their lifestyle.

Recently, Fuchs and coworkers took advantage of a whole-genome shotgun sequencing approach to assemble, annotate, and analyze the sequence of strain W22703 of *Y. enterocolitica *[22]. Their research study provided valuable information on the strategies utilized by *Y. enterocolitica* to cope with its environment. Wang et al. [23] sequenced the complete genome of *Y. enterocolitica *strain 3/O:9 and strain 8081 (1B/O:8); the comparison of the genome sequences of these two strains indicated that these two strains' different pathogenicity may have been a result of completely separate evolutionary events. Recent efforts by Batzilla et al. [24] to compare the complete genome of *Y. enterocolitica palearctic* serobiotype O:3/4 to the available genome of *Y. enterocolitica *ssp. *enterocolitica* 8081 O:8/1B indicated that gene loss and acquisition during evolution through mobile genetic elements could be the contributing factor to differentiate pathogenic bacteria from apathogenic bacteria of the same species. *Y. enterocolitica *is a heterogeneous bacterial species with a complex life cycle encompassing aquatic and biological environments. Further genome sequencing and analysis will help us to learn more about the evolution of *Y. enterocolitica *strains and provide the necessary information for the development of molecular-based detection methods for *Yersinia *in food systems.

Rouillard and Gulari developed a pangenomic oligonucletide microarray probe set database called OligoArrayDb [25]. OligoArrayDb was designed for most of the sequenced genomes that are not covered by commercial catalog arrays. Based on their algorithm of analysis, the *Y. enterocolitica *strain 8081 genome, a total of 4137 transcripts and containing 11821 oligonucletides, were chosen to represent the *Y. enterocolitica *strain 8081 transcriptome. Among these oloigonucleotides, 11251 are considered to be fully specific to their targets. This microarray probe set can be accessed through the website at http://berry.engin.umich.edu/oligoarraydb/index.html. 

### 2.2. Microarray Analysis

The dominant application of microarrays has been in measuring gene expression in different biological conditions [26–28]. Other important microarray applications include comparative genomic hybridization [29], chromatin immunoprecipitation [30], mutation detection [31], genotyping [32], and array-mediated localized cell transfection [33, 34]. Microarray technology involves the placement of user-defined oligonucleotide probes in specific locations on a solid matrix such as glass or filters. The concept behind all microarrays is the precise placement of DNA fragments at high density on the solid support, so that they can act as molecular detectors. There are many variations of this method based on the solid matrix used and more importantly, the different types of DNA fragments on the array, including cDNA, oligonucleotides, and genomic fragments. Currently, there are three main types of microarrays: filter arrays [35], spotted glass slide arrays [36], and in situ synthesized oligonucluetide arrays [37] available for research purposes. 

Following the hybridization of target DNA sequences to probes on the solid matrix, fluorescence-based detection can be used to monitor binding signal and be recorded. Along with the rapid development of microarray technologies, there has been an unprecedented amassing of data collected by academic institutes, as well as industrial organizations. Software applications can be used to conduct data analysis and greatly facilitate the data analysis process. There are many open-source, public-domain, and commercial solutions for data storage, analysis, management, and exportation. Most of the applications are being updated frequently to keep current with the new demands from research. Several applications have been released that integrate data acquisition, processing, analysis, and exportation [25, 38]. The commercial GeneSifter (http://www.geospiza.com/Products/AnalysisEdition.shtml), the academic GenMAPP (http://genmapp.org/), and the open-source BASE (http://base.thep.lu.se/) aim to provide the functionalities for data analysis. Some software applications also provide comprehensive solutions for image analysis and data extraction. Most recent software applications for microarray data analysis are listed in Table 1. 

Microarray methods provide an effective way of distinguishing between nonspecific and target product formation following PCR amplification of target DNA sequences from the samples. Amplification methods have been used previously in combination with microarray technology for the detection of *Y. pestis*. Huang et al. [39] were able to specifically detect *Y. pestis *from *Y. enterocolitica *and* Y. pseudotuberculosis* using a microarray method combined with PCR amplification. Myers and coworkers [40] developed a microarray chip combined with PCR amplification for detection and characterization of four virulence genes (*virF*, *ail*, *yst*, and *blaA*) in *Y. enterocolitica.* They were able to identify *Y. enterocolitica* from adulterated pasteurized whole milk using this approach. Ikeda et al. [41] were able to detect three foodborne bacteria: *Salmonella enterica* serovar Enteritidis, *Y. enterocolitica,* and *Bacillus cereus* in fresh vegetables using a DNA microarray method. Kim et al. [42] used comparative genomics to select 70-mer ologonucleotide probes specific for 11 major foodborne pathogens for use in microarray analysis. All of these studies have demonstrated that genome sequencing and DNA microarray analysis have a powerful application in detection of pathogenic *Yersinia* in food systems.

### 2.3. Immunoassay

Antibodies have been used for many years to type bacterial isolates serologically [43–45]. The development of the enzyme-linked immunosorbent assay (ELISA) introduced highly sensitive tests for specific targets with great reliability. Key advantages of ELISA are its ease of use, flexibility, and low cost. The highly specific nature of antibodies, especially monoclonal antibody (MAbs), and the simplicity and versatility of antigen-antibody reactions have facilitated the design of a variety of assays, and they comprise the largest group of molecular biological methods being used in foodborne pathogen detection [46–48]. 

*Yersinia pestis *is antigenically homogenous, but *Y. enterocolitica *and* Y. pseudotuberculosis* have multiple O and H antigens [49]. ELISA kits for detection of *Y. enterocolitica *are commercially available for the detection of the O antigen; for example, Mabs anti-O:3 and -O:9 can be purchased from LifeSpan BioSciences for research purposes. 

Other methods for evaluating immunological binding events include fluorescence-based microscopy and surface plasma resonance. A commonly used field-portable immunoassay is the lateral flow disposable membrane technology. This technology is designed for threshold or qualitative testing. Advantages of this format include low-cost, portability, room-temperature stability and no need for specialized equipment and only minimal user training is required [50]. 

Multiplexing format immunoassays, suitable for the simultaneous evaluation of multiple targets in a sample, can be developed to increase the analytical productivity and drastically reduce analysis costs and sample and reagent consumption. For the low-multiplexing assay without automation, quantitative PCR, ELISA, or Western blotting allow multiple targets to be measured simultaneously and quantitatively. For the high multiplexing OMIC technologies, microarrays, SELDI, and LC/MS allow measurement of several hundred potential targets, but the output is essentially qualitative. There are two main multiplex immunoassay formats currently being applied widely in research: (1) protein attached microarrays [51, 52] and (2) bead-based microarrays [53, 54]. Magliulo et al. [55] developed a simple, multiplexed sandwich chemiluminescent enzyme immunoassay for the simultaneous detection of four of the major foodborne pathogens: *Escherichia coli* O157:H7, *Y. enterocolitica, Salmonella *Typhimurium, and *Listeria monocytogenes*. The accuracy and precision of this method were comparable to those achievable with the conventional culturing methodology yet detection was completed significantly faster than in traditional practices. 

Protein microarray is a novel technology for quickly detecting and identifying proteins [56]. A protein detecting microarray comprises many different affinity reagents arrayed at high spatial density on a solid support. Each agent captures its target protein from a complex mixture, and the captured proteins are subsequently identified. For routine detection purposes, there is substantial benefit to be gained from using protein microarray technology. In principle, thousands of proteins can be spotted on a single slide, enabling one to interrogate simultaneously the presence of many different proteins with minimal sample consumption. Furthermore, hundreds of copies of an array can be manufactured, enabling the same proteins to be probed repeatedly with many different molecules from different samples. Rucker and coworkers have successfully developed antibody-based microarray techniques for the multiplexed detection of cholera toxin *β*-subunit, diphtheria toxin, anthrax lethal factor, and protective antigen, *Staphyloccus aureus* enterotoxin B, and tetanus toxin C fragment from spiked samples [57]. Li et al. used a protein microarray spotting with 149 *Y. pestis* proteins to profile antibody responses to a *Y. pestis* live vaccine [58]. With the continuing innovation for this technology, some limitations need to be addressed, as well. For protein detection microarrays, the cross-reactivity of affinity reagents need to be assessed and reduced. For a protein function microarray, the purity and integrity of the proteins need to be determined.

Immunoassays have an important role in the diagnosis and monitoring of diseases in routine-based pathological laboratories. However, immunoassay sensitivity and potential cross-reactivity should be carefully considered in comparing detection methods. Nucleic-acid-based technology may be a suitable alternative for a range of molecular targets traditionally detected by immunoassays [59].

### 2.4. Next-Generation Sequencing

DNA sequencing is one of the most important molecular tools in any life sciences field [12, 60]. Over the past 30 years, there has been more than a millionfold improvement in the rate of sequence generation with the progression from radio-labeled products using slab gels to fluorescent products and capillary electrophoresis to next-generation sequencing technologies [60]. According to Stratton, in the future, the cost of sequencing may drop greatly where, for example, the costs of sequencing whole cancer genomes can drop to US$1000. Routine sequencing in a clinical, diagnostic setting will then become feasible [60]. 

Next Generation Sequencing (NGS) technology has been adopted as a sequencing tool for quite some time [61–63]. This sequencing technology has the following features: massively paralleled sequencing without electrophoresis, samples need to be prepared and amplified, and extensive usage of computer resources. NGS can be categorized into (1) microelectrophoretic methods, (2) sequencing by hybridization, (3) real-time observation of single molecules, and (4) cyclic array sequencing [64]. 

There are significant differences between conventional sequencing technologies and NGS platforms in terms of sequencing chemistry, application, and cost [64, 65]. The comparison of major NGS technologies and conventional sequencing technologies is summarized in Table 2. The applications of conventional sequencing using the Sanger approach are suitable for small-scale sequencing within the kilobase to megabase range [66, 67]. The requirements of a Sanger sequencing approach include major costs such as robotic support of reagents, processing of multiple samples in either 96- or 384-well formats, and regular maintenance of capillary-based sequencers. NGS has fewer infrastructure requirements than the Sanger sequencing approach. Among the NGS platforms, there are important differences that may result in advantages with respect to specific applications (Table 2). Some applications may be more tolerant of short read lengths than others. The accuracy, as well as the specific error distributions of individual technologies, may also be relevant [68–71]. 

The diversity and advancement of NGS technology pose challenges for bioinformaticists to address, such as the issues of alignment, assembly, sequence scoring, data storage, and data release. Two major computational approaches are performed with NGS reads, assembly and alignment. The assembly approach is performed when no reference genome exists for the DNA sequenced, such as in the case of a genetically uncharacterized pathogen. Assembly algorithms take sequence reads, align overlapping sections, and generate longer length contigs, which serve as the scaffold for genome assembly, and subsequent alignments [72–74]. Alignment process is used to determine the best match between sequence reads and the reference sequence. To accommodate the large number of reads generated by NGS, a number of new alignment algorithms have been developed. These algorithms share the characteristic that alignment is performed in a multistep or heuristic approach in which the first phase consists of converting either the sequence reads or the reference sequence into an index of shorter length sequences, which are given read identifiers [75–77]. Postalignment, programs generate key information including the number of aligned reads, a list of sequence variants relative to the reference, and the percentage of reads containing the variant. A variety of software applications have been developed using these algorithms and are being widely utilized by researchers. Some of the popular tools are listed in Table 3.

Some of the key applications for NGS include (1) whole genome *de novo* sequencing and single nucleotide polymorphism (SNP) discovery [63, 68, 78], (2) mapping of structural rearrangements and transformation events [79], (3) expressed sequence tags (ESTs) or serial analysis of gene expression [80], (4) transcriptome assembly for gene discovery and transcription profiling [81], (5) large-scale analysis of DNA methylation [82], (6) genome-wide mapping of DNA-protein interactions [83], (7) confirmatory sequencing in gene cloning [84], and (8) genome-map-based cloning [85]. 

Cummings and coworkers [86] used the SOLiD system (Applied Biosystems, Calif) to conduct parallel microbial whole genome typing to detect strain-specific polymorphism in *Bacillus anthracis* and *Y. pestis*. Their research results suggested the possibility of using NGS technology during a forensic or epidemiological investigation facilitating high-resolution strain tracking. Morelli et al. [87] utilized both conventional sequencing and NGS technologies to identify patterns of global phylogenetic diversity through the comparison of 17 whole genomes of *Y. pestis *isolates from global sources. Chen et al. used NGS technology to obtain and compare sequencing data from 3 pathogenic and 8 nonpathogenic members of the *Yersinia* genus [88]. They identified 100 regions within the genome of *Y. enterocolitica* that represented potential candidates for the design of nucleotide sequence-based assays for detection of the pathogen.

NGS has fundamentally impacted various fields of biological research, including food safety. This technology can be transitioned into the clinical diagnostic area. Similar to the development of microarray technology, the challenges will shift from mastering this technology to the question of how best to extract meaningful biological or clinical information from the large amount of data generated by this technology.

## 3. Summary

Food has often been suggested to be the main source of yersiniosis. Current methods to detect foodborne pathogens rely traditionally on culture media to select and propagate viable cells in foods. However, the isolation rates of pathogenic *Y. enterocolitica* have been low, which may be due to the limited sensitivity of the culture methods. The new advancement of the current technologies will provide cheaper, more accurate, and faster methods to identify pathogenic *Yersinia* in food systems during a food-related pathogenic crisis. 

Despite better detection efficiencies, results derived using molecular biology methods can be affected by the various food matrices, the presence of normal bacterial flora, and interferences by some of the food ingredients. It still remains a challenge to develop methods that are rapid, sensitive, and specific in detection of foodborne pathogens. With the improvements in sample preparation, data analysis, and testing procedures, molecular detection techniques will likely continue to simplify and increase the speed of detection while simultaneously improving the sensitivity and specificity for tracking pathogens in food matrices. 

The molecular-based detection methods discussed, above all, have advantages and limitations. Even use of the same detection method such as real-time PCR approach, different target genes used for the assay can limit the detection sensitivity. The detection range can vary from single colony forming unit (CFU) per ml to 10^3^ CFU/mL. Similarly, the lateral flow stripe requires a relatively high concentration of target organisms between 10^7^ CFU/mL to 10^10^ CFU/mL. Due to the limitations of individual detection methods, the combination with other techniques should be used for verification to ensure adequate specificity and sensitivity of the detection results. Combining with other methods also enhances the performance of individual assays. Owing to the complex variables in food analysis, most molecular-based methods for detecting foodborne pathogens are used for screening purposes, where the positive results need to be confirmed by cultural methods.

## Figures and Tables

**Table 1:** Current software applications for microarray data analysis.

Software	Application	Provider	Platform	Web link
Array Designer	Primer design for microarray construction	Premier Biosoft International	Windows Linux	http://www.premierbiosoft.com/dnamicroarray/index.html

ArrayMiner	Analysis tool for microarray gene expression data	Optimal Design	Mac OS Windows	http://www.optimaldesign.com/ArrayMiner/ArrayMiner.htm

ArrayTrack	Database solution for managing, analyzing, and interpreting microarray gene expression data	National Center for Toxicological Research U.S. Food and Drug Administration	Web-based	

				http://www.fda.gov/ScienceResearch/BioinformaticsTools/
				Arraytrack/default.htm



ArrayVision	Automated analysis of macro- and microarrays	GE Healthcare	Windows	http://www.gelifesciences.com/aptrix/upp01077.nsf/Content/
				Products?OpenDocument&ParentId=957136

BAMarray	Detecting differentially expressed genes from microarray data using Bayesian analysis	Case Western Reserve University	Mac OS Windows Linux	http://www.bamarray.com/default.htm

BASE	Database solution for the massive amounts of data generated by microarray analysis	Lund University	Web-based	http://base.thep.lu.se/

Cluster	Perform a variety of types of cluster analysis and other types of processing on large microarray datasets	University of Tokyo	Mac OS Windows Linux/Unix	http://bonsai.hgc.jp/~mdehoon/software/cluster/software.htm

GenePattern	Gene expression analysis tools	Broad Institute, MIT	Web-based	http://www.broadinstitute.org/cancer/software/genepattern/desc/
				expression.html

GeneSifter	Tools for exploring the statistically significant interplay of the data with factors of biological relevance to understand the expression pattern in microarray data.	Geospiza Inc.	Web-based	http://www.geospiza.com/Products/AnalysisEdition.shtml

GenMAPP	Tools for visualizing data from gene expression experiments in the context of biological pathways.	Gladstone Institute, University of California at San Francisco	Windows	http://genmapp.org/

GenMaths XT	Analysis of high density microarrays and gene chips	Applied Maths	Windows	http://www.applied-maths.com/genemaths/genemaths.htm

Genowiz	A comprehensive multi platform software for microarray data analysis	Ocimum Biosolutions	Mac OS Windows Linux/Unix	
				http://www3.ocimumbio.com/data-analysis-insights/
				analytical-tools/genowiz/


Microarray tools	Including: a Comparative Genomic Hybridization (CGH) and expression microarray data analysis, data management and export system	J. Craig Venter Institute	Windows Linux/Unix	http://www.jcvi.org/cms/research/software/#c622/

Partek Genomics Suite	Statistical analysis and data mining tools to facilitate powerful and intuitive exploratory data analysis	Partek Incorporated	Windows Linux/Unix	http://www.partek.com/partekgs/

TreeArrange and Treeps	Software for displaying and manipulating hierarchical clustered data	University of Waterloo	Windows Linux/Unix	http://monod.uwaterloo.ca/downloads/treearrange/

waviCGH	For the analysis and visualization of array-CGH data	Spanish National Cancer Center, Bioinformatics Unit	Web-based	http://wavi.bioinfo.cnio.es/

**Table 2:** Comparison of major next generation DNA sequencing technologies and conventional sequencing.

Platform	Application	Sequencing chemistry	Read length (bases)	Throughput per run (Gb)	Read per run (million)	Throughput per 24 hr (Gb)	Raw accuracy Range (%)	Cost Pe Mb ($)
ABI 3730	(1) Complement *de novo* assemblies for high-quality assembly of complex genomes; (2) Custom sequencing (3) Targeted resequencing for polymorphism discovery and genotyping	Sanger Dideoxy	800	0.00008	0.000096	0.00064	99.0 to 99.999	4000

ABI SOLID 5500	(1) Whole genome SNP discovery; (2) Transcriptome assembly and expression profiling; (3) Whole methylome resequencing	Sequencing by ligation	60 × 2	310	5167	45	99.0 to 99.9	0.05

Illumina HiSeq	(1) Whole genome SNP discovery; (2) Transcriptome assembly and expression profiling; (3) Whole methylome resequencing; (4) Bacterial and megaplasmid *de novo* assembly	Sequencing by synthesis	100 × 2	600	6000	75	96.2 to 99.7	0.02

Life Technologies Ion Torrent	(1) Whole methylome resequencing; (2) Bacterial and megaplasmids *de novo *assembly; (3) Sequencing quality control; (4) Sequencing requirement lower complexity	pH meter	200	0.2	1	2.4	>99.0	0.5

Roche 454	(1) *De novo* assemblies of complex genomes; (2) Metagenomics; (3) Analysis of large structural variations	Pyrosequencing	600	0.8	1	0.5	96.0 to 97.0	8

**Table 3:** Software applications for NGS analysis.

Software	Categories	Sequencing file format compatibility	Created by	Operating platform	Web link
ABySS	Assembly	FASTA FASTQ QSEQ SAM BAM	Jared Simpson et al. Michael Smith Genome Sciences Centre	Mac OS Linux POSIX	http://www.bcgsc.ca/platform/bioinfo/software/abyss/

Edena	Assembly	FASTQ	David Hernandez University of Geneva Hospitals	Windows Linux	http://www.genomic.ch/edena.php/

Exonerate	Alignment	FASTA	Guy Slater and Ewan Birney European Bioinformatics Institute	Windows Linux Unix	http://www.ebi.ac.uk/~guy/exonerate/

Maq	Alignment	FASTA FASTQ Illumina Bustard & Gerald Illumina ELAND	Heng Li	Windows Linx	http://maq.sourceforge.net/

Mosaik	Alignment	FASTA FASTQ Illumina Bustard & Gerald SRF	Michael Stromberg and Gabor Marth Boston College	Mac OS Windows Linux	http://code.google.com/p/mosaik-aligner/

Phrap/ Cross_match/ Swat	Alignment	FASTA	Phil Green, Brent Ewing and David Gordon University of Washington	Mac OS Windows Linux	http://www.phrap.org/phredphrapconsed.html

PyroBayes	Base Caller	SFF	Aaron Quinlan et al. Boston College	Linux	http://bioinformatics.bc.edu/marthlab/PyroBayes/

SHARCGS	Assembly	Illumina Bustard & Gerald Illumina ELAND	Juliane Dohm et al. Max Planck Institute	Linux	http://sharcgs.molgen.mpg.de/

SHRiMP	Alignment	FASTA FASTQ SAM Illumina Bustard & Gerald	Michael Brudno and Stephen Rumble University of Toronto	Mac OS Linux	http://compbio.cs.toronto.edu/shrimp/

SOAP	Alignment Burrows-Wheeler	Illumina Bustard & Gerald Illumina ELAND	Ruiqing Li et al. Beijing Genomics Institute	Unix	http://soap.genomics.org.cn/

SSAHA2	Alignment Smith-Waterman	FASTA FASTQ SAM Illumina Bustard & Gerald	The Wellcome Trust Sanger Institute	Mac OS Linux	http://www.sanger.ac.uk/resources/software/ssaha2/

SSAKE	Assembly	FASTA	Rene Warren et al. Michael Smith Genome Sciences Centre	Linux	http://www.bcgsc.ca/platform/bioinfo/software/ssake/

VCAKE	Assembly k-mer extension	FASTA	William Jeck et al.	Mac OS Linux	http://vcake.sourceforge.net/

Velvet	Assembly	FASTA FASTQ Illumina Bustard & Gerald Illumina ELAND	Daniel Zerbino et al.	Mac OS Linux Cygwin	http://www.ebi.ac.uk/~zerbino/velvet/
